# Forty years of the Brazilian response to HIV: reflections on the need for a programmatic shift and policy as a common good

**DOI:** 10.1590/0102-311XEN199423

**Published:** 2023-12-08

**Authors:** Alexandre Grangeiro, Dulce Ferraz, Laio Magno, Eliana Miura Zucchi, Márcia Thereza Couto, Ines Dourado

**Affiliations:** 1 Faculdade de Medicina, Universidade de São Paulo, São Paulo, Brasil.; 2 Diretoria Regional de Brasília, Fundação Oswaldo Cruz, Brasília, Brasil.; 3 Instituto de Saúde Coletiva, Universidade Federal da Bahia, Salvador, Brasil.; 4 Departamento de Ciências da Vida, Universidade do Estado da Bahia, Salvador, Brasil.; 5 Programa de Pós-graduação em Saúde Coletiva, Universidade Católica de Santos, Santos, Brasil.

We appreciate the comments by Barreira & Alencar ^1^ and Paiva & Ayres ^2^, pointing to more effective ways of responding to HIV in Brazil. In 2023, which marks the 40th anniversary of the first actions against HIV in Brazil, initiated in 1983 by the Somos Group, we aim to highlight the need to resume a discussion with Brazilian society to “awake” HIV policy and reformulate it according to the new socio-epidemiological reality.

In the first article of this debate ^3^, we addressed a challenge: although the variety of existing prevention methods allow us to aim for the elimination of HIV, the persistence of access barriers, combined with substantial generational changes that altered the forms of sexual encounters and collective mobilization have made the determinants of the HIV epidemic in Brazil more complex and intertwined. In this scenario, we agree with Paiva & Ayres ^2^ (p. 1) that policy needs to be “*debated and constructed in the public space as a common good, subsidized by scientific evidence and guided by an ethical-political examination*”. We also agree with Barreira & Alencar that, at the moment, we need, above all, a leap forward in our combating programs.

Therefore, a critical look at prevention policies is essential, raising the question: which actions should be strengthened? Which actions should be created? And even further, which actions should be abandoned because they no longer make sense? Given Barreira & Alencar’s criticism of the proposal to prioritize the supply of methods, what have been the results of insisting on offering a core package for HIV prevention, treating each method as equals? Why would we insist on this strategy knowing that the effectiveness and acceptability of each method is different or that individual choices are crossed by inequalities? If we seek a science-based response, we need to go beyond the evidence of effectiveness and apply the consolidated knowledge of the psychosocial, economic, and cultural dimensions that interfere with prevention to policy. It was, in fact, a partial understanding of science ₋ overvaluing efficacy data ₋ that subsidized what Barreira & Alencar called the overvaluation of condoms. Thus, our defense of the hierarchization of preventive supply does not oppose the right of access and the obligation to make methods widely available, but proposes a guide to the organization of policy, guidance for professionals and managers, and clear information for society.

We cannot fall into the trap, in line with Paiva & Ayres, of underestimating the importance of pre-exposure prophylaxis (PrEP) due to the economic interests of the pharmaceutical industry. We must reclaim, in both ethical and economic terms, the role of Brazilian politics in the fair, universal and equitable incorporation of health technologies that have been proven to benefit populations ^4^; as well as remember that vaccines and prophylaxis, before being forms of any kind of control, are instruments of human agency in the face of epidemics and diseases. Thus, prioritizing PrEP should also be understood as strengthening the health industrial complex, technical-scientific production, and, consequently, the regulatory capacity of Brazil and the Global South.

This leads us to another point: to clarify to Paiva & Ayres that we do not separate PrEP from the sexual scene, but we recognize the potential of this prophylaxis to allow preventive decisions to be made and implemented outside of it. This does not mean separating PrEP from the scene, since our experience with users of this prophylaxis shows that the meanings constructed for relationships and scenes also condition preventive decisions, such as using PrEP for greater sexual agency ^5^. Therefore, continuing to invest in “literacy for prevention” is essential, aiming at psychosocial emancipation, allowing people to find ways to use the potential of each technology to ensure protection in its various scenes. Moreover, PrEP has created new conditions for sexual scenes, in many cases eliminating negotiations about condom use, according to studies of gay men, who often publicly declare their use of PrEP, especially on dating apps ^6^. We are thus facing a new era of the epidemic, in which the most affected communities have reformulated their sexual practices, for example, by incorporating condoms, seroadaptive practices, and now PrEP and treatment as prevention (TasP). Once again, we must learn from these communities and think about how to use these innovations in public policy to benefit even more vulnerable populations, such as gay and trans adolescents.

Reiterating our understanding that only strong and democratic policies will reverse the growth of the epidemic among young people, we did not see the need to extrapolate prevention from the institutions represented by health services and professionals to a “deinstitutionalization of prevention”. We would prefer to expand institutionalization, recognizing that sustainable community actions require policies that promote funding, technical support, and articulation with the health system. We highlight this fact due to some paradoxes: although community actions have played ₋ and still play ₋ a key role in the response to the epidemic, their national and international funding has drastically decreased since the 2000s. Despite the continuous nature of community actions, public support has continued for more than 40 years to be provided by calls for proposals for specific projects; and although the forms of social organization have multiplied, the public-community policy relationship continues to prioritize formal non-governmental organizations.

Moreover, it is important to recognize that universalization and equity in the Brazilian system are absolutely dependent on health services and professionals; and that the Brazilian Unified National Health System (SUS) is organized based on actions in the community and in the territory, with a focus on integration between health surveillance and primary care. In this sense, we once again draw attention to the lower prioritization of HIV and the decrease in federal funding for decentralized government programs, with the weakening and sometimes disappearance of these programs in the states and municipalities.

Therefore, Barreira & Alencar’s proposals for a leap forward in our programs, deinstitutionalization, and the establishment of prevention at the earliest opportunity, in a simple and agile way, will only be achieved, in our opinion, with a profound reformulation of funding policies, aimed at institutionalization, expansion, and articulation between government and community responses.

Similarly, we highlight the role of education, in synergy with what Barreira and Alencar called the right to information and Paiva & Ayres called literacy of young people and with young people. From our perspective, this process cannot ignore the School Health Program. In line with the Brazilian Common Core National Curriculum, this is the most likely environment for building life projects, considering singularity and an expanded and plural understanding of youth. Moreover, in this collaborative learning environment, adolescents can take ownership of the production and circulation of knowledge and critically develop repertoires on health, preventive methods, and human rights.

Finally, we draw attention to the fundamental point that unites our vision with Paiva & Aires and Barreira & Alencar: tackling the epidemic is a common good, built by the community. In the field of HIV, we often use the term community to refer to social organizations and social movements. However, the changes in recent years mentioned in the first article of this debate ^3^ ₋ the organization of young people into collectives, the expansion of the LGBTQIA+ movement, new ways of expressing gender and sexuality ₋ seem to force us to redefine this understanding. A promising way is to adopt the intersectional perspective as the basis of the response articulated together with the communities, as they organize themselves and create meanings for their resistance. The community that built the successful Brazilian response consisted of governments, civil society, and academics. This is therefore the community that needs to be rescued and strengthened. The spaces for social participation and dialogue with scientific knowledge have been seriously weakened in recent years, a reflection of authoritarianism. We understand that these spaces are being reconstructed and here we want to value this process and push the reflection on the need to reinvent forms of participation, health policies and practices.
